# Case report: Psychedelic-induced seizures captured by intracranial electrocorticography

**DOI:** 10.3389/fneur.2023.1214969

**Published:** 2023-06-29

**Authors:** Benjamin N. Blond, Emmanuelle A. D. Schindler

**Affiliations:** ^1^Department of Neurology, Health Science Center, Renaissance School of Medicine at Stony Brook University, Stony Brook, NY, United States; ^2^Department of Neurology, Yale School of Medicine, New Haven, CT, United States; ^3^Neurology Service, VA Connecticut Healthcare System, West Haven, CT, United States

**Keywords:** psilocybin, psychedelics, seizures, epilepsy, case report

## Abstract

Classic psychedelics are currently re-emerging as therapeutic agents with unique clinical benefits; however, it is also important to recognize the adverse effects of this drug class. While the risk of seizures with this drug class is known, the literature is lacking in detail. We present a case of psychedelic mushroom-induced seizures in a person with refractory right temporal lobe epilepsy implanted with a responsive neurostimulation (RNS) system. A large increase in typical seizure frequency coincided with the ingestion of a large dose of the mushrooms. This is the first reported case of electrographically confirmed seizures associated with classic psychedelic drug use. With the surge of research and movements toward the clinical application of classic psychedelic compounds, the risk for drug-induced seizures should be considered, including factors such as a history of epilepsy and drug doses and regimens.

## Introduction

Classic psychedelics are currently re-emerging as therapeutic agents with unique clinical benefits. While this presents a significant opportunity, it is important to temper enthusiasm for this drug class with an appropriate knowledge of potential adverse effects (1). This case report describes a person with epilepsy who had a significant exacerbation of seizures correlating with ingesting psychedelic mushrooms. This case is unique in that the patient has refractory right temporal epilepsy for which he has been treated with a responsive neurostimulation (RNS) system, and the recordings from this device provide objective documentation of typical seizures in association with the psychedelic exposure. This is an important addition to the literature on the increased risk of seizures with psychedelic drugs, which is currently lacking in detail. This case has implications for the accuracy of prior subjective seizure reporting with psychedelic use and the mechanisms of triggering seizures.

## Case report

The patient is a 31-year-old man who has had refractory focal epilepsy for 21 years with focal seizures without impaired awareness characterized by a sensation of warmth, diaphoresis, and overwhelming anxiety and focal to bilateral tonic–clonic seizures. These seizures correlated with right temporal onsets on intracranial electroencephalography (EEG). He was implanted with an RNS, as Wada and neuropsychological testing indicated an increased risk of deficits with resection. RNS was implanted bilaterally as the weaker than expected function in the left temporal region raised suspicion that it may also be an epileptogenic focus, but the RNS has since confirmed unilateral right temporal epilepsy over 2 years of monitoring. Morbidity significantly improved, but he continues to have several focal seizures without impaired awareness per week. The RNS is programmed to detect ictal onset patterns. When these patterns persist for a preset duration (30 s for this patient), they are termed long episodes. These long episodes typically represent electrographic seizures or prolonged epileptiform activity (2), and in this patient, the long episodes recorded by the RNS correlate well with seizures on electrocorticography and the patient's overall clinical reports.

**Figure 1:**
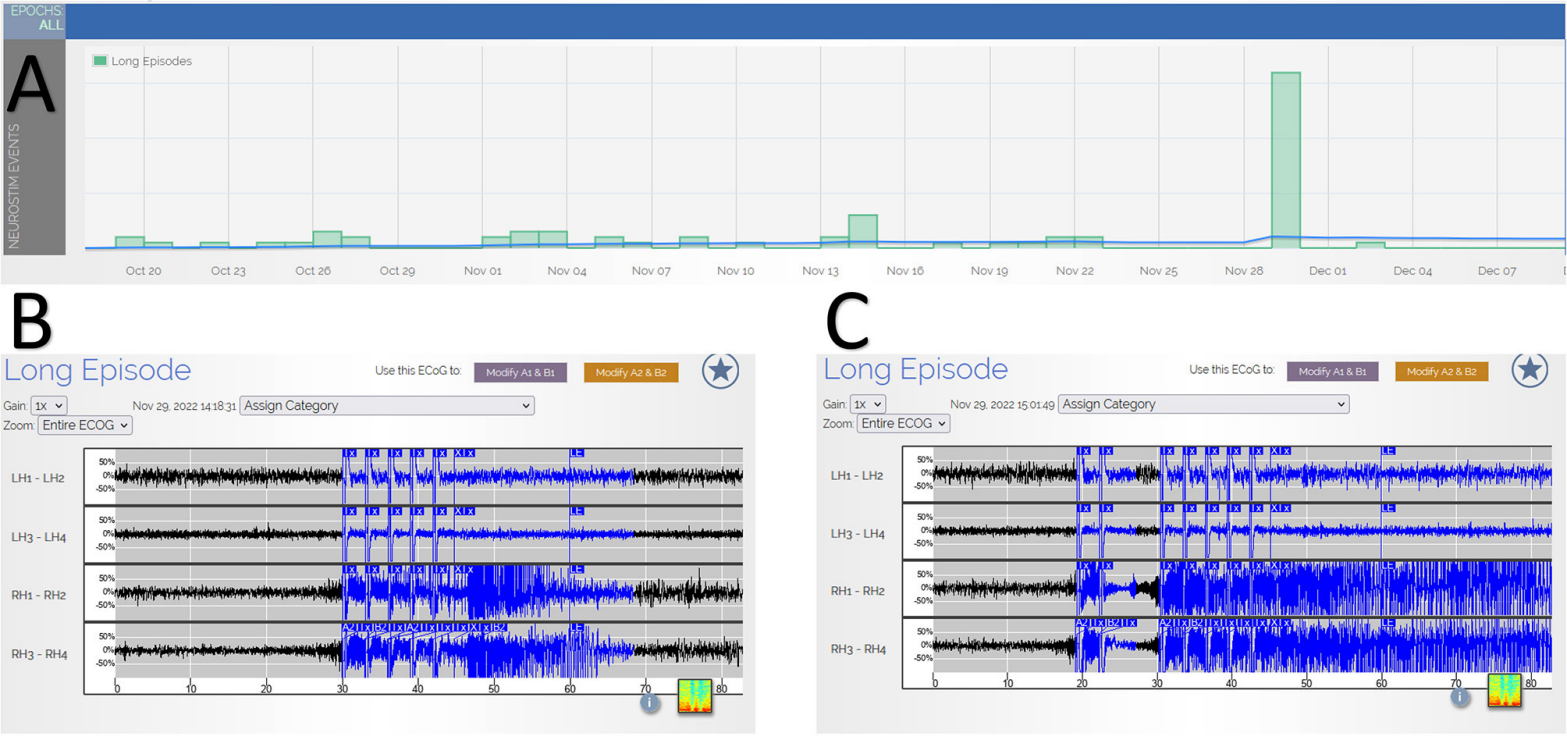
**(Panel A)** shows RNS tracking of long episodes over time, which demonstrates a significant increase in long episodes on the day of the higher dose psychedelic mushroom use with 32 long episodes. The x-axis is calendar dates demonstrating the timeline of the occurrence of the episodes. The y-axis is the number of long episodes, with each box representing 10 long episodes. **(Panels B, C)** provide examples of electrocorticography recordings of two of the long episodes, demonstrating typical seizures. The x-axis is time in seconds, and the y-axis is the relative voltage for each channel. LH, left hippocampus; RH, right hippocampus; numbers 1–4 refer to the depth electrode contact.

**Figure 2:**
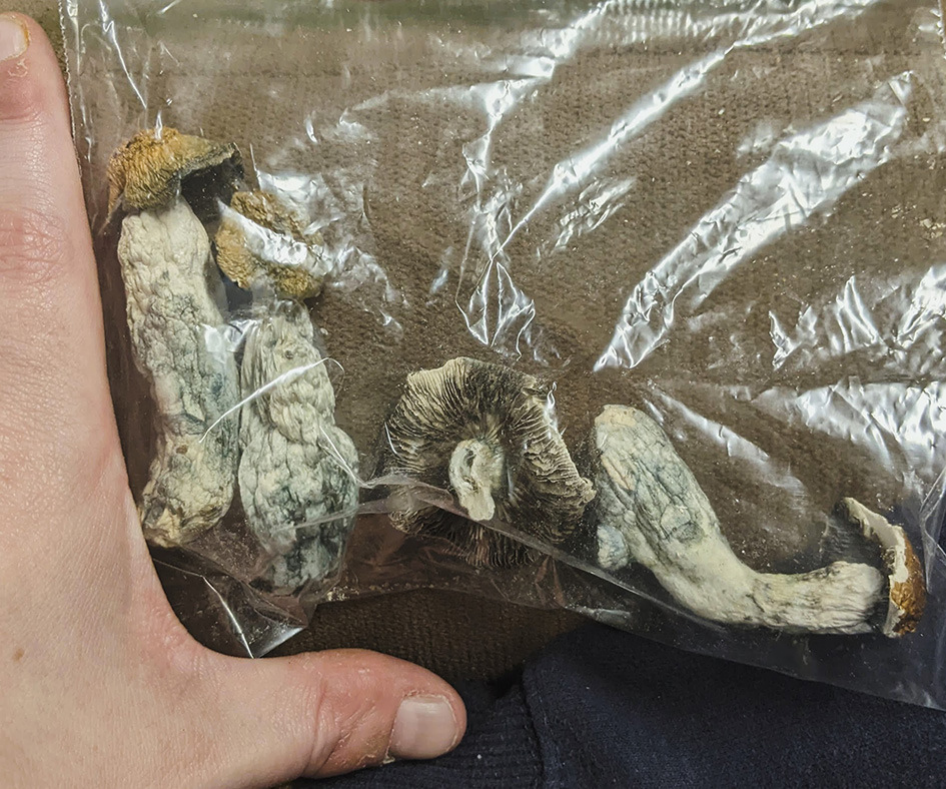
A photograph showing the dried mushrooms consumed by the patient, which are identifiable as *Psilocybe cubensis*. He fully consumed the second mushroom from the left on the first day (the day of the seizures), estimated to be 2.5–3.0 g (~20 mg pure psilocybin). On subsequent days, he took smaller amounts, not more than 1.5 g (~10 mg pure psilocybin) at a time, over 1 week.

During a routine clinic visit, the review of RNS data demonstrated a significant outlier with 32 long episodes in 1 day (Figure 1). This is substantially larger than his typical long episode counts of 0-2 on the same RNS settings. In total, 5 out of the 32 long episodes had full EEG tracings recorded (the number of full recordings is limited by the memory capacity of the RNS), and all of these episodes were consistent with electrographic seizures. The patient identified this as the day he used psychedelic mushrooms recreationally (Figure 2). He had used these in the past before developing epilepsy but had not yet used them since. On the first day, he consumed a whole mushroom and felt high, and he described feeling symptoms similar to when his seizures occur but with calmness instead of anxiety. Feeling this high was too intense, so in subsequent days, he took smaller amounts of mushroom, using up his remaining supply over the following week. He had one additional seizure during those days, consistent with a return to his baseline pattern. He measured the smaller amounts of mushroom on a scale, taking no more than 1.5 g of dried mushroom at a time. Based on these weights, he retrospectively estimated that he had taken 2.5–3.0 g of dried mushroom on the first day. The patient denied other potential factors that could have triggered the seizure cluster. He denied any significant changes in his life circumstances, dietary changes, sleep deprivation, or missed doses of anti-seizure medications (levetiracetam and pregabalin). He also reported no change in his baseline daily marijuana and weekly alcohol use and denied the use of other controlled substances.

## Discussion

This case highlights a significant exacerbation of RNS-documented seizures in a person with epilepsy in association with psychedelic mushroom use. We are unaware of any other such cases in which seizures were confirmed on intracranial electrocorticography. Although this patient continues to have uncontrolled epilepsy and seizure clusters, the cluster of 32 long episodes on the RNS was a clear outlier in his history. The mushrooms he consumed are identifiable as *Psilocybe cubensis*, which contain the classic psychedelic, psilocybin. Classic psychedelics are 5-hydroxytryptamine 2A receptor agonists that produce acute sensory and perceptual alterations and include such compounds as psilocybin, lysergic acid diethylamide (LSD), and dimethyltryptamine (DMT). A number of psychotropic drugs have fallen under the term psychedelic, including 3,4-methylenedioxymethamphetamine (MDMA, ecstasy), ketamine, and cannabis, but these drugs are pharmacologically distinct. Classic psychedelics have a known association with seizures (3, 4), although the data are limited. Poison control centers have reported seizures in 0.78% of psilocybin (*n* = 5,883), 4% of LSD (*n* = 3,554) (5), and 2.2% of ayahuasca (containing DMT and beta-carbolines; *n* = 538) (6) cases. Preliminary reports suggest a high rate of seizures (40%, *n* = 20) with the N-methoxybenzyl class of synthetic psychedelics (7). Seizures related to classic psychedelics may be more common with a history of epilepsy. Out of 613 respondents who endorsed lifetime psychedelic use, 9 reported having associated seizures. In total, 5 out of these 9 individuals reported a personal history of epilepsy and 7 reported a family history of epilepsy (8). We note that these individuals may be more likely to report seizures as they have prior experience in identifying them. Interestingly, our patient did not recognize he was having seizures, as he attributed his sensations to the psilocybin high. Subjective seizure reporting by individuals may be unreliable due to acute perceptual alterations while intoxicated. More objective reporting of clinical features of seizures (e.g., convulsive activity) is limited by the availability of observers and whether they are also partaking in drug use. These various factors may result in a general under-reporting of psychedelic-induced seizures. RNS documentation of seizures serves to eliminate uncertainty in correctly identifying seizures in people with epilepsy, particularly when there is a lack of awareness or observable changes in behavior that could be misidentified as seizures.

The cause of seizures from classic psychedelics is not known, although the activation of monoamine systems may be involved. The explanation is not a simple one, however, as fenfluramine, a monoamine-releasing agent, was recently approved for the treatment of Dravet and Lennox Gastaut Syndromes (9, 10). Earlier experimental models in baboons revealed both pro- and anti-seizure properties after intravenous administration of classic psychedelic and related drugs (11, 12). While general excitement about the broad therapeutic potential of classic psychedelic drugs might raise the question about clinical *benefit* in epilepsy, given the preponderance of evidence showing an increased risk of seizures, particularly in those with epilepsy, and lack of convincing evidence for anti-seizure effects, such an idea may be hazardous. Interestingly, in the present case, the patient had his typical focal seizures recorded on intracranial electrocorticography in association with psychedelic use. This supports the concept of a lowering of the seizure threshold for typical seizures rather than inducing seizures through an alternate pathway. The literature on provoked seizures is often unclear on the mechanism involved and whether it differs among provoking substances or between people with or without epilepsy. This single case cannot inform on whether provoked seizures are more or less likely with psychedelic use compared with other substances or the mechanisms involved but suggests interesting areas for future research.

The present case also suggests that the exacerbation of seizures may be dose related. Our patient had multiple seizures with a larger dose of psilocybin mushroom but did not have any change in his baseline seizure frequency while using lower doses. Clear data on drug purity and dosing in humans having psychedelic-induced seizures are largely unavailable due to the illicit nature of these substances (3–5). Research in animals has also been limited and subjected to the additional challenge of appropriate scaling of dose from animals to humans. In a famous historical case, the fatal overdose of an Asian elephant due to seizure involved the questionable dose conversion of LSD among animal species (13). Based on the species of mushroom our patient consumed, the higher dose he took contained approximately 20 mg psilocybin and the lower dose contained approximately 10 mg psilocybin (14). Human clinical trials of psychedelic-assisted therapy in mental health use a range of 25 to 30 mg of psilocybin. These doses induce strong psychedelic and mystical experiences, which are felt to be required for therapeutic effects in these psychiatric conditions. In contrast, studies in headache disorders, including migraine and cluster headache, only use 10 mg psilocybin (15, 16), and acute drug experiences are not correlated with therapeutic outcomes. Regardless of the dose used, the risk of seizure in psychedelic clinical trials is reduced through rigorous screening procedures that exclude participants with serious medical conditions, such as epilepsy, or prior severe reactions to classic psychedelics or other recreational drugs. While our case suggests possible risk with higher doses of psilocybin, the safety of lower doses has not been established to date. This point is particularly important, given the emerging practice of chronic use of low doses (i.e., “microdosing”), which is outside the bounds of our established understanding of and experience with classic psychedelic drugs. Our patient only took a few low doses of psilocybin mushroom over a week, though there are reports of patients with chronic pain conditions using low doses of psilocybin mushrooms chronically over months to a year (17). The consequences of such practices over the long term have yet to be determined.

This report has several limitations. This is an observation restricted to one individual with 1 week of psychedelic mushroom use that occurred in a home setting. Given the patient's uncontrolled epilepsy, it is reasonable to question whether the seizure cluster could have occurred spontaneously. We find this unlikely given the objective documentation of the cluster via intracranial electrocorticography with long term comparison data failing to show other such clusters as a part of this person's epilepsy (There were 2 years and 2 months of data at the time the cluster was identified and reviewed). We can neither independently verify the dosing of psychedelic mushrooms nor his concomitant amounts of alcohol and marijuana, but the patient was helpful in providing his best recollections and estimates. Our consideration of a possible dose-related response would be strengthened by additional data, but in the interest of his safety, we counseled the patient to avoid further psychedelic substances. During his clinic visit, a decision was made to increase the neurostimulation charge density as part of the ongoing treatment of epilepsy. While this was important for the patient's clinical care, it confounds any potential analysis of a longer-term effect of psilocybin on the patient's epilepsy.

This case is noteworthy as it provides evidence of typical focal seizures via intracranial electrocorticography in association with psilocybin-containing mushroom use. As classic psychedelics have re-emerged as potentially promising therapeutics for neuropsychiatric, headache, and chronic pain disorders, raising this safety issue is timely. The public, particularly people with epilepsy, should be cautioned about the risks of seizures from psychedelic drugs. Unfortunately, much of the information produced for popular consumption is imprecise, over-promises and sidesteps safety considerations. Clinical providers and researchers should also be cautioned about the risk of seizures with these substances and should carefully consider such factors as epilepsy history and drug dosing and chronicity when discussing psychedelics with patients.

## Patient's perspective

The patient's perspective on this case was integrated into the text above to provide appropriate context, but it is worth reiterating. The patient was not aware that he had seizures on the day that he used the higher dose of psilocybin. When we were reviewing his RNS data in the clinic, the topic was raised on that day with a particularly severe cluster of seizures and whether anything out of the ordinary occurred. On looking at the date, the patient reported that this was the day he had ingested psychedelic mushrooms and commented that he had wondered whether this might affect his brain activity. He believed that the feelings he had were a part of the high of the drug experience. Since the experience from the first dose was too intense, in future days, he was careful to take a smaller amount of the mushrooms.

Upon learning of the association between seizures and psychedelic mushroom use, the patient was appreciative of the level of care involved in our RNS clinic and how the device can provide such diagnostic data. He appreciates that there is an effort to think about his life as a whole and integrate his epilepsy care into that larger picture. He was enthusiastic about the opportunity of sharing his story in the medical literature if it could help other people. Currently, he is being careful to avoid higher dosage use of psychedelics.

## Data availability statement

The original contributions presented in the study are included in the article/supplementary material, further inquiries can be directed to the corresponding author.

## Ethics statement

Written informed consent was obtained from the individual(s) for the publication of any potentially identifiable images or data included in this article.

## Author contributions

BB is the physician caring for the patient in the case report, performed the clinical assessment, and wrote the first draft of the manuscript. BB and ES equally contributed to the overall conception of the article, literature review and revisions of the manuscript. Both authors approve of the final manuscript.
